# Ectopic Papillary Carcinoma in the Midline Neck Accompanied by a Benign Thyroid Gland

**DOI:** 10.1155/2019/9172942

**Published:** 2019-01-10

**Authors:** Lauran Evans, SeHoon Park, Christie Elliott, Courtney Garrett

**Affiliations:** ^1^University of Nevada, Reno School of Medicine, Reno, NV, USA; ^2^Renown Regional Medical Center, Reno, NV, USA

## Abstract

Ectopic thyroid tissue can deposit anywhere in the body. There are several cases reporting papillary thyroid carcinoma (PTC) arising from ectopic tissues; however, these cases largely presented with primary PTC within the native thyroid gland as well. Alternatively, some cases report of PTC found solely in an ectopic thyroglossal duct cyst, but reports of isolated malignancy in other types of ectopic thyroid tissue with normal native tissue are sparse throughout the literature. Here, we present an unusual case of PTC in the midline anterior neck that does not appear to be consistent with a thyroglossal duct cyst, accompanied by a completely benign native thyroid gland, of which only few cases have been reported.

## 1. Introduction

Ectopic thyroid tissue is a common variant of normal thyroid anatomy and is usually an incidental finding during autopsy or surgery. The most common location for this tissue to deposit is within a thyroglossal duct cyst, though ectopic thyroid tissue can be found anywhere between the tongue and the diaphragm [1]. For example, several cases reporting thyroid tissue in the thorax can be found throughout the literature [1].

Ectopic thyroid tissue is subject to malignant transformation. Similar to that of the native thyroid gland, the most common malignancy found is PTC [1]. With the exception of the lingual thyroid, ectopic thyroid malignancies are often euthyroid and asymptomatic. Thus, they are usually diagnosed when they enlarge enough to cause clinical symptoms via mass effect [1].

Differentiating isolated ectopic thyroid tissue malignancy from metastases of native thyroid tissue has presented a challenge, as the developmental mechanism of these ectopic thyroid malignancies is poorly understood [1, 2]. One circulating hypothesis in the literature posits that ectopic thyroid carcinomas may represent metastases of native malignancy. However, sporadic cases in which ectopic thyroid carcinoma presents with normal native tissue support an alternative hypothesis that ectopic thyroid tissue may develop malignancies independently from the native thyroid gland [1]. Several cases of ectopic thyroid carcinoma with absent native malignancy are reported in literature with presentations in the thyroglossal duct cyst [3, 4]; submandibular [5], unilateral [6], and bilateral [7] cervical masses; branchial cleft cyst [8]; and mediastinum [2, 9, 10]. In this case report, we present an unusual instance of a midline ectopic thyroid malignancy, with absence of features characteristic of a thyroglossal duct cyst, in the context of normal-appearing native thyroid tissue.

## 2. Case Presentation

**Figure 1:**
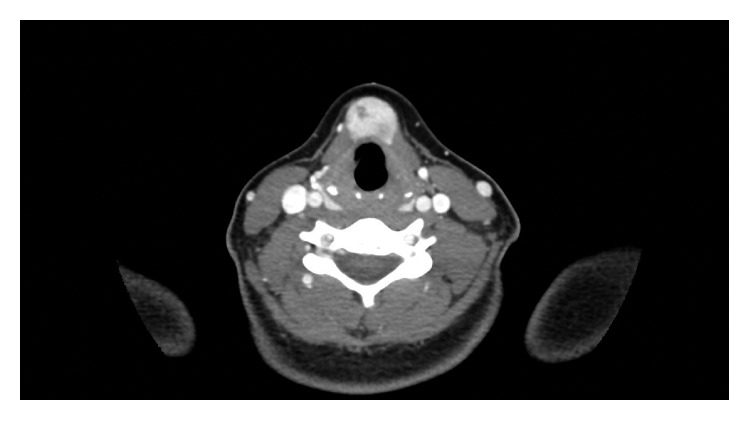
CT neck at the level of vertebra C4 showing a 1.6 × 2.0 × 2.9 cm, enhancing, elliptically shaped mass located in the midline, within the anterior soft tissues of the neck.

A 17-year-old female presented to the otolaryngologist complaining of rapidly enlarging neck swelling noticed three months earlier after a wisdom tooth extraction. She was given antibiotics for a presumed dental or thyroglossal duct cyst infection without improvement. She was subsequently referred for an otolaryngology consult for further evaluation. The patient denied neck pain, dysphagia, difficulty breathing, or weight loss. She has no history of significant radiation exposure or family history of thyroid malignancy. Past medical history was significant for chronic otitis media status post bilateral myringotomy tubes 9 years ago but had otherwise been healthy. Physical exam revealed a well-circumscribed “golf ball” shaped mass in the midline of the neck between the thyroid cartilage and hyoid bone that elevated with swallowing. Tympanic membranes showed mild scarring, but the remainder of the exam was unremarkable. Suspecting a thyroglossal duct cyst, the otolaryngologist planned for a Sistrunk procedure preceded by a CT neck to evaluate the extent of the mass and visualize the thyroid gland. The CT neck showed a 1.6 × 2.0 × 2.9 cm, enhancing, elliptically shaped mass located within the anterior soft tissues of the neck inferior to the hyoid bone, anterior to the hypopharynx and glottis. The mass enhanced similarly to the thyroid tissue, read by the radiologist as suspicious for ectopic thyroid (Figure 1). There were no enlarged lymph nodes, and the thyroid gland appeared normal.

**Figure 2:**
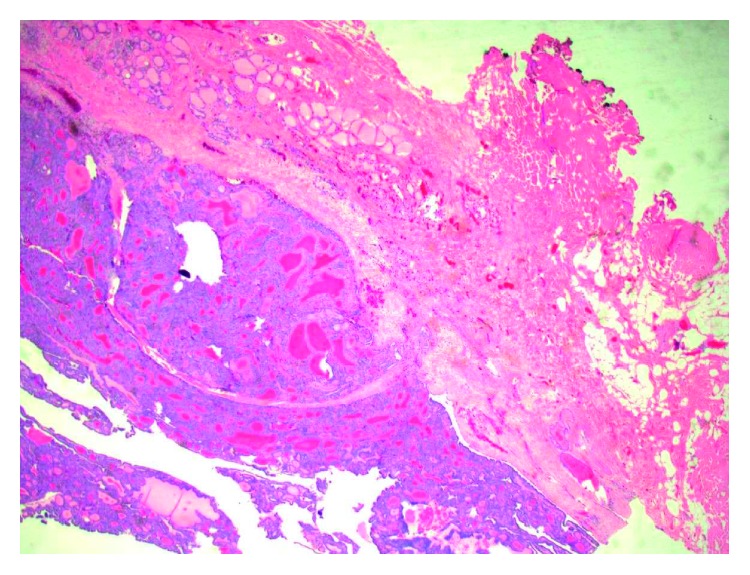
H&E stain at 4x magnification of anterior neck mass demonstrating epithelial capsule without cilia surrounding papillary architecture and few normal thyroid components. No evidence of cystic components is seen. The histological stain shows that the anterior neck mass is consistent with an ectopic papillary thyroid carcinoma.

The patient underwent excision of the neck mass without complication. During the surgery, the mass was noted to sit atop the thyroid cartilage; no obvious tracts were seen. Grossly, the specimen was described as a 3 × 2 × 1.8 cm, irregular, pink-tan friable nodule with focally hemorrhagic surfaces. A frozen section showed papillary architecture adjacent to normal thyroid architecture with both tissue types surrounded by an epithelial capsule that was devoid of cilia (Figure 2). No cystic components were seen. Cytology revealed intranuclear inclusions, nuclear grooves, and ground-glass nuclei. The stated abnormalities were determined by two collaborating pathologists to be highly suggestive of PTC arising from a focus of ectopic thyroid tissue. Margins were determined to be uninvolved. The lymph node was <1 mm and was negative for the disease.

Thyroid function tests, complete metabolic panel, and complete blood count were within normal limits, except for a thyroglobulin level of 48 ng/ml. Because of the risk for concurrent native thyroid malignancy, the patient underwent an uneventful total thyroidectomy. The specimen revealed a native thyroid with a normal architecture. A surrounding lymph node was resected and also shown to be benign.

Postoperatively, the patient developed symptomatic hypocalcemia that was successfully managed with calcium and vitamin D. She was discharged home in stable condition on thyroxine, calcium, and vitamin D. One month postoperatively, she was asymptomatic with normalized calcium levels and thyroid tests. She will return to the endocrinologist in 3 months for a thyroid uptake scan and potential radioactive iodine if residual disease is found.

## 3. Discussion and Conclusion

Ectopic thyroid tissue can undergo malignant transformation and is classically accompanied by a similar transformation of the native thyroid gland. Several cases have reported a benign thyroid with ectopic PTC within thyroglossal duct cysts or specific regions of the upper body but none involved the anterior neck.

On initial presentation, this patient's mass was believed to simply represent a thyroglossal duct cyst because of the sudden onset, patient's age, mass location, and commonality of the condition. Unexpectedly, PTC was found without evidence of a thyroglossal duct cyst. In the absence of tracts, cystic components, and capsular epithelial cilia, it is suggested that this PTC arose from ectopic thyroid tissue in the anterior neck rather than a thyroglossal duct remnant. To our knowledge, this is the first time an isolated midline cervical ectopic thyroid malignancy lacking the characteristics of a thyroglossal duct cyst has been described in literature. The rapid growth of this malignant mass was also quite unusual.

The majority of ectopic thyroid malignancies present with a corresponding malignancy in the native tissue. The current standard of treatment in this situation is to recommend a total thyroidectomy. The rarity of an isolated pathology and the lack of tools for early detection have contributed to our poor understanding of development of ectopic thyroid malignancies. Further analysis and a larger sample size are needed, however, to weigh the risk and benefit of total thyroidectomy. Ultimately, a normal thyroid gland should not exclude the diagnosis of a malignancy in ectopic thyroid tissue that may be present in other areas. By reporting this case, we hope to inform surgeons of the various unusual presentations ectopic thyroid gland malignancies can take, leading to more efficient diagnosis and treatment.
